# SAMHD1 as the Potential Link Between SARS-CoV-2 Infection and Neurological Complications

**DOI:** 10.3389/fneur.2020.562913

**Published:** 2020-09-25

**Authors:** Aiza Khan, Consolato Sergi

**Affiliations:** ^1^Department of Laboratory Medicine and Pathology, University of Albert Hospital, Edmonton, AB, Canada; ^2^Department of Pediatrics, Stollery Children's Hospital, University of Alberta Hospital, Edmonton, AB, Canada

**Keywords:** COVID-19, neuroinvasion, ACE2, SAR-CoV2, string, bioinformatics, neurodegeneration, prognosis

## Abstract

The recent pandemic of coronavirus infectious illness 2019 (COVID19) triggered by SARS-CoV-2 has rapidly spread around the globe, generating in severe events an acute, highly lethal pneumonia and death. In the past two hitherto similar CoVs, the severe acute respiratory syndrome CoV (SARS-CoV-1) and Middle East respiratory syndrome CoV (MERS-CoV) also gained universal attention as they produced clinical symptoms similar to those of SARS-CoV-2 utilizing angiotensin-converting enzyme 2 (ACE2) receptor and dipeptidyl peptidase 4 (DPP4) to go into the cells. COVID-19 may also present with overtly neurological symptoms. The proper understanding of the expression and dissemination of ACE2 in central and peripheral nerve systems is crucial to understand better the neurological morbidity caused by COVID-19. Using the STRING bioinformatic tool and references through text mining tools associated to Coronaviruses, we identified SAMHD1 as the probable link to neurological symptoms. Paralleled to the response to influenza A virus and, specifically, respiratory syncytial virus, SARS-CoV-2 evokes a response that needs robust induction of a subclass of cytokines, including the Type I and, obviously, Type III interferons as well as a few chemokines. We correlate ACE2 to the pathogenesis and neurologic complications of COVID-19 and found that SAMHD1 links to NF-κB pathway. No correlation was found with other molecules associated with Coronavirus infection, including ADAR, BST2, IRF3, IFITM3, ISG15, MX1, MX2, RNASEL, RSAD2, and VPRBP. We suggest that SAMHD1 is the molecule that may be behind the mechanisms of the neurological complications associated with COVID-19.

## Introduction

Coronaviruses (CoVs), which belong to *Coronaviridae* family, are enveloped RNA viruses that have been associated with respiratory and extra-respiratory (e.g., enteric and neurological) diseases in various animal species (1). Human coronaviruses (HCoVs) are commonly known to be accountable for both lower and upper infections of the respiratory tract (2). In the last two decades, SARS-CoV-1 and MERS-CoV caused severe acute respiratory syndrome coronavirus and the Middle East respiratory syndrome, which triggered a large-scale public health response (3). In December 2019, another novel coronavirus SARS-CoV-2-, labeled astempestively as COVID 19, emerged and has been the cause of a worldwide pandemic (4). SARS-CoV-2 is found to have 75–80% remarkably genomic similarity to SARS-CoV-1 and 50% genomic similarity to the Middle East Respiratory Syndrome coronavirus (MERS-CoV) (5). All three of these viruses belong to the family of β-coronaviruses, with bats being the possible common reservoir (6). These β-coronaviruses typically produce respiratory and gastrointestinal symptoms in human and animal hosts, respectively (5–7). However, there is accumulating evidence that in addition to these two systems, coronaviruses may conquer the central nervous system (CNS) as well causing diseases affecting the neurological system. In fact, patients of COVID-19 have been reported to exhibit some symptoms of Guillain Barre syndrome (GBS) as well as other neurological diseases (8–10). Pondering the high similarity between SARS-CoV-1 and SARS-CoV-2, some preliminary studies are suggesting that the potential CNS invasion may partially be responsible for the mortality (other than morbidity) associated with COVID-19 infection and have some late neurologic *sequelae* (8, 11).

## COVID-19 Virus Obtain Entry In The Interior of The Host Cells Via ACE2 Receptor

These enveloped viruses harbor a positive-strand RNA genome with dimensions of up to 31 kb, which is representative of the largest known genome including all RNA viruses (9). This genome consists of specific genes coding numerous structural and non-structural proteins. Along With these proteins, the Spike or S protein possesses a unique biological significance (9). It has been associated with properties such as tropism and its modulation (9, 10). It is important to remember that the S units of coronaviruses are crucial in aiming for a cellular receptor that mediates infection in the target cells (10). Similar to SARS, angiotensin-converting enzyme 2 (ACE2) has been recognized as the functional receptor for SARS-CoV-2 as well (7, 12). The intracellular penetration of SARS-CoV-1 and MERS-CoV utilize the ACE2 receptor and DPP4, respectively. With the mRNA programming several other proteins, SARS-CoV-2, uses S1 to enable the attachment of the virion to the cellular membrane by networking with the host ACE2 receptor (13). It has also been elucidated that spike proteins belonging to all three CoV are not identical, even though they are considered similar. The ACE2 binding affinity identified in the 2019-nCoV spike protein ectodomain is roughly 10–20-fold higher as compared to that of the SARS-CoV spike protein, thus justifying the higher required affinity of the COVID-19 spike protein to the human counterpart (ACE2) receptor (13). ACE2, which is produced in multiple human organs, including human upper airway epithelia, lung, kidney, and gut parenchyma as well as vascular endothelia, is also present in the brain (13–16).

The brain expression of ACE2 is indicative of the fact that SARS-CoV-2 may obviously cause some symptoms of neurological type via direct or indirect mechanisms (13). Investigations have reported the occurrance of various neurological symptoms. In the following section, we discuss the distribution of ACE2 receptors in the brain and attempt to address potential short term and long-term implications of brain involvement of this infection using STRING as a bioinformatic tool (17).

## Clinical Characteristics

COVID-19 patients have been delineated to exhibit symptoms such as fever, myalgia, diarrhea, and cough (18). Typical clinical presentation of SARS-CoV-2 consists of fever, dry cough, and progressive respiratory distress at the onset of illness, which may lead to lethal pneumonia. It has been estimated that more than half of patients exhibiting dyspnea end up needing intensive care (8, 18, 19). Respiratory failure, infections, and cardiovascular events are the leading cause of mortality (8, 11, 20). Importantly, recent studies suggest that, in COVID-19 patients, in some cases, neurological symptoms occur as well (14, 19, 21). Furthermore, when present, neurological manifestation of COVID-19 infection has been categorized as CNS and peripheral nervous system (PNS) symptoms. CNS symptoms consist of headache, dizziness, impaired consciousness, and even, acute cerebrovascular disease (14, 22–26). PNS symptoms may consist of hyposmia, hypoplasia, neuralgia, and hypogeusia. Moreover, very recently, a patient with viral encephalitis has been reported (26). Overall, these findings support the notion that SARS-CoV-2 does possess neuroinvasive properties. It is crucial to gain knowledge of the molecule, which is central in its neurotropism.

## Neurotropism is One Common Feature of CoVs

Several studies have elaborated on the neuroinvasive nature of CoVs (27). Animal studies have identified the brain as a primary focus organ for infection in rodents (mice) that are transgenic for the SARS-CoV receptor (ACE2) (28, 29). Additionally, further studies of this model elucidated that that the virus may enter the brain via the unique path of the olfactory bulb, causing infection with a rapid, spread (transneuronal) to connected cerebral areas. Death of the animal was assumed to be from non-functionality and/or destruction of infected neurons, notably those located in cardiorespiratory centers located in the *medulla oblongata*. Studies demonstrated that neurons are a highly vulnerable target for SARS-CoV (28). Neurological damage has been confirmed in the infection of coronavirus, such as in SARS-CoV and MERS-CoV, as well as, currently, SARS-CoV-2 (30). Studies on MERS-CoV demonstrated that low inoculum dosages of MERS-CoV viral particles were discovered only in the brain of mice, while none in the lung, indicative of the fact that mortality may have been associated with infection in the CNS (31–33). In particular, the brainstem has been shown to be severely infected by SARS-CoV (34, 35) or MERS-CoV (20). Additionally, the autopsy findings in patients who suffered from SARS-CoV infections have demonstrated robust evidence of the existence of SARS-CoV by SARS genome sequences being restricted to the cytoplasm of various neurons in the hypothalamus and brain cortex. Also, edema (cytologic hydropic degeneration) and scattered red disintegration of the neurons was reported in autopsies as well as suggestive of the fact that these viruses are capable of invading the nervous system (34).

## Neuroinvasion of SARS-CoV-2 and SAMHD1

The idea has been put forth concerning how specific viruses enter into the nervous system. Numerous studies suggest that at first, certain CoVs colonize peripheral nerve terminals, and subsequently enter CNS via a synapse-connected path (35–38). Some other CoVs have been reported to enter the brain through trans-synaptic transfer (8). As far as SARS-CoV and MERS-CoV are concerned, experimental studies of animal models showed that SARS-CoV and MERS-CoV, when given through the nose, possibly invade the *cerebrum* via the olfactory nerves, spreading to some regions of the brain including thalamus and brainstem (8, 20, 34). Notably, it has been suggested that ACE2 olfactory (epithelial) support cells and stem cells produce ACE2 genes, as do cells located in the epithelium of the nasal respiratory tract in patients afflicted with COVID-19, which may show some parallels with attention-deficit/hyperactivity disorder (39–41). So far, in the light of the evidence provided enough, it is conceivable that SARS-CoV-2 is neuroinvasive, and it uses ACE2 as receptors to penetrate the cell (13). We target the ACE2 distribution in the brain, based on literature and discuss various associated pathways, which can potentially cause mortality in COVID-19 infected patients.

Evaluating protein -protein interactions can be tremendously essential in understanding the underlying biological mechanisms of any disease (42). Protein-protein interactions possess an integral role in several biological functions which include metabolic and signaling pathways' regulation, replication of DNA, and immunological recognition, among others (43). In addition to providing a reasonable analysis of interactions, STRING also offers a full set of images of the interaction network. It utilizes domains, pathways, and Gene Ontology annotations in order to support the functional enrichment of network proteins (44, 45). It is a bioinformatic tool and Text-Mining tools belonging to the Swiss Institute of Bioinformatics (SIB), European Molecular Biology Laboratory (EMBL), and Novo Nordisk Foundation Center Protein Research (CPR). Therefore, we used the bioinformatic tool STRING and references through text mining tools that has been correlated with Coronaviruses. Using STRING, we discovered SAMHD1, which is the acronyme for “sterile-α-motif and HD domain-containing protein 1” as the probable link to neurological symptoms. Equated to the response to influenza A virus and, specifically, respiratory syncytial virus, SARS-CoV-2 provokes a response that lacks induction of a subgroup of cytokines, including types I and III interferons as well as a few chemokines. In the setting of pathogenesis and neurologic complications of COVID-19, we could not find that SAMHD1 had links to ADAR, BST2, IRF3, IFITM3, ISG15, MX1, MX2, RNASEL, RSAD2, and VPRBP. *SAMHD1* plays a role in the control of the innate immune response. The coded protein is upregulated in reaction to viral infection and may be involved in the intercession of tumor necrosis factor (TNF)-α pro-inflammatory responses.

## Potential Host Responses To COVID-19

So far, we have established the potential invasion of the brain by COVID 19. At this point, It is also essential to consider the host response to this virus from the therapeutic perspective (2). It is commonly known that one core function of the innate immune system is to find virologic infections and induce antiviral effectors to prevent the spread of disease and activates antigen-specific adaptive response (46). As indicated above, COVID 19 lung tissue shows that SARS-CoV-2 evokes a distinctive response of transcriptional type, which lacks Types I and III interferons (IFN-I and IFN-III) expression (47). Along with this, there is an induction of well-categorized direct effectors of the innate immunologic response, including SAMHD1, MX1, IFITM3, and TRIM25, as well as the induction of viral sensors of RNA type such as RIG-I and the OAS1-3 genes (47). It is to note that previous studies on CoVs have also indicated that CoVs prevent IFN induction at transcription level as well as subsequent antiviral gene activation (48). Typically speaking, the innate response consists of signaling cascades that are activated upon the detection of the foreign pathogens in the host (49). Generally, during a viral infection, cytoplasmic sensors of protein type, such as RIG-I (retinoic acid-inducible gene I) and MDA5 (melanoma differentiation-associated protein 5), bind to viral single-stranded RNA and/or, remarkably, double-stranded RNA (dsRNA) and signal through a protein called MAVS (mitochondrial antiviral signaling protein) (42, 43). Initiation of MAVS results in the subsequent activation of TBK1/IKKε, which are TANK-binding kinase 1 (TBK1) as well as the homolog IκB kinase (IKK) epsilon (IKKε, originally IKKi), two kinases which phosphorylate IRF3 (Interferon Regulatory Factor 3) to induce its dimerization, and import into the nucleus (43). TBK1/IKKε have been studied profusely in relation to their functions in inducing the type I interferon response. Nuclear IRF3, along with several other proteins, leads to transcription and induction of type I IFN to warn neighboring cells of the infection (44). As soon as IRF3 induces transcription of IFN-I, this potent cytokine is emitted from the cells. The NF-κB pathway is also remarkably activated via these mediators. Activation of classical NF-κB is essential for a successful immune response as well as for the survival and proliferation of cells (45). Interestingly, exogenous SAMHD1 expression in cells and, alternatively, SAMHD1 reconstitution in knockout cells have been shown to quell NF-κB activation and IFN-I induction, as specifically reported (50).

**Figure 1:**
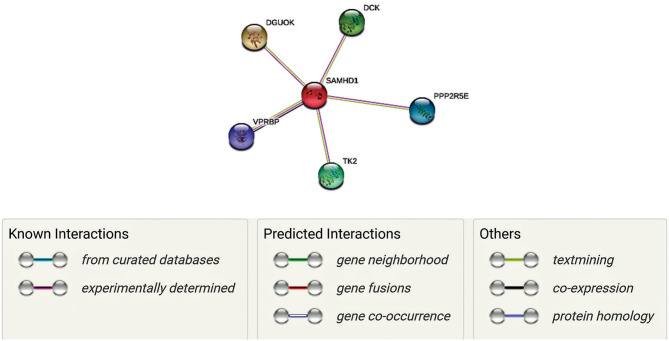
Network representation of proteins linked to SAMHD1 using version 11 of STRING. Each gene has a unique function as well as interacts with SAMDH1 in maintaining the concentrations of deoxyribonucleoside triphosphates (dNTPs) by regulating synthesis and degradation in DNA synthesis. Mutations of some of these genes have been correlated with AGs (Aicardi-Goutières syndrome (AGS) is a rare disease, characterized by genetically determined early-onset progressive encephalopathy). Thus, we hypothesize that these interactions are essential in the context of neurological symptoms associated with COVID 19.

SAMHD1 is a vertebrate protein. It is engaged in the control of cellular deoxyribonucleoside triphosphates (dNTP) pool by catalyzing the hydrolysis of dNTP into 20-deoxynucleoside and triphosphate products (51). SAMDH1 has been reported to be a key mechanism of cell proliferation and is an essential player in dNTP homeostasis. SAMHD1 is a dNTP hydrolase which reduces the concentration of intracellular dNTP pools (52, 53). It has been suggested that the inhibitory effect of SAMHD1 on HIV-1 replication is due to its dNTPase action, or it has also been suggested that SAMHD1, or an associated protein, may limit HIV-1 replication through its RNase activity (54). In Figure 1 is shown an interactive panel of a number of proteins linked to SAMHD1 using version 11 of STRING. In mammalian cells, the rates of dNTPs are thought to be controlled by coaction of synthesis and degradation. In this process, synthesis of dNTPs involves two corridors, (1) *de novo* synthesis of dNDPs by ribonucleotide reductase (RNR) in the cytosol followed by (2) phosphorylation to dNTPs by a nucleoside-diphosphate kinase (55). Rescue of deoxyribonucleosides occurs by two parallel sets of deoxy-nucleoside and -nucleotide kinases in both the mitochondria and cytosol (55).

Along with SAMDH1, DCK (deoxycytidine kinase), DGUOK (deoxyguanosine kinase), PPP2R5E (Serine/threonine-protein phosphatase 2A 56 kDa regulatory subunit epsilon isoform), VPRBP (Vpr (HIV-1) binding protein), and TK2 (thymidine kinase 2) may play unique roles in this process. In fact, VPRBP has a role in cell growth, as it regulates the G_1_ phase of cell cycle and is crucial for the achievement of DNA replication in the S phase (56, 57). PPP2R5E has been demonstrated to have multiple signaling pathways and is vital during early development (58). While TK2, DGOUK, and DCK are involved in the deoxynucleoside salvage pathway (59–61), it is to note that VprBP has been linked with SAMDH1 in the context of its antiviral activity against HIV. Mutations of TK2 and DGOUK have been linked with SAMDH1 in the context of AGS elated neurological symptoms (56, 59). Interestingly, regulation of their function and the DGOUK mutations have also been known to cause a specific type of mitochondrial encephalopathy (61). Thus, it is conceivable that a SAMDH1 and its interactions with these proteins, as well as the potential impairment of these interactions, are essential in the antiviral response as well as neurological symptoms associated with might be involved in COVID 19.

Current evidence suggests SAMHD1 is an effective antiviral restriction factor suitably targeting numerous other medically relevant viruses (62). However, it is to note that the study on CHIKV and ZIKV reported that SAMHD1 might be proviral as it promotes their replication. This study found that Vpx/VLP-mediated SAMHD1 deterioration leads to a conspicuous decrease in the replication and, notably, virion production by both viruses. While the same treatment reduced the replication of CHIKV (Chikungunya virus, an alphavirus, replicon, it was also observed that the over-expression of SAMHD1 facilitated CHIKV and ZIKV (Zika virus) replication (63). It is worth mentioning that unlike HIV-1 and HBV, in CHIKV and ZIKV, the reverse transcription step is not needed for their replication, hence could be the reason for such contrasting results. Also, unlike HIV, in CHIKV and ZIKV the presence of dNTP (deoxynucleoside triphosphate) is not required. Furthermore, it has also been demonstrated that SAMHD1 interacts with the IKKε and IFN regulatory factor 7 (IRF7), thus causes the suppression of the IFN-I induction pathway by reducing IKKε-mediated IRF7 phosphorylation. Also, SAMHD1 inhibits NF-κB activation by interacting with NF-κB1/2, thereby reducing phosphorylation of the NF-κB inhibitory protein IκB (50, 63).

Mechanistically, SAMHD1 promotes an inhibiton of the NF-κB activation by interacting with NF-κB1/2 and decreasing phosphorylation of the NF-κB inhibitory protein IκBα. Also, SAMHD1 interacts with IKKε and IRF7, leading to the suppression of the IFN-I induction pathway by actively reducing IKKε -mediated IRF7 phosphorylation. The intimate interactions of endogenous SAMHD1 with NF-κB and IFN-I pathway proteins have been specifically validated in both human monocytic cells and primary macrophages (63).

Notably CHIKV and ZIKV both show neurological symptoms as well (63). It is important to recall that numerous patients with Zika fever have also been reported to have symptoms of Guillain-Barre Syndrome (GBS) (64). Hence it can be assumed that SAMDH1 might be a commonality and a causative factor among these viral infections. Along with them, SAMDH1 has been associated with several other disorders that show signs and symptoms of neurological involvement (65–68). These include Aicardi-Goutieres syndrome (AGS), which is an autoimmune condition. Aicardi-Goutieres syndrome (AGS), which is caused by irregular type I IFN responses, results in physical and intellectual disabilities. AGS patients end up with mild to severe mental or physical impairments. Additionally, *SAMHD1* mutation has been reported to play essential roles in immunoregulation and cerebral vascular homeostasis. It has been thought that cerebrovascular events (e.g., stroke) associated with specific mutations in *SAMHD1* extend the phenotypic spectrum of AGS (69). Various inflammatory vasculopathies of the brain that may lead to cerebrovascular stenoses and early stroke have been correlated with SAMDH1 as well (70). Moreover, SAMHD1 expression has been associated with neurological symptoms of HIV as well (71). Also, mutations of *SAMDH1* have been correlated with the pathogenesis of neurogenerative disorder as well (64). Overall, these findings suggest that SAMDH1 is involved in various diseases related to the brain.

Like other innate immunity proteins, SAMDH1 shows a crucial role in shaping innate and adaptive immunological responses to protect animals against viruses by disrupting the virus' life cycle (52). In response to that, viruses generate proteins that bind these host factors and alter their activity. This setting leads to an evolutionary conflictual event as immunity and virus proteins exquisitely adapt to prevent and restore binding, respectively (52, 54). One such adaptation has been reported to occur in a region of *SAMHD1* that controls its activity, which influences its enzymatic properties suggesting that evolutionary conflictual eventhas engaged modulation of SAMHD1 regulation and function and has swayed both SAMHD1's dNTPase and antiviral actions (52, 54, 63).

There is evidence of SAMDH1 being both antiviral and proviral (50, 63). Thus, in the perspective of COVID 19 infection, it is profoundly essential to improve the knowledge of how SAMHD1 executes its activity in CNS as well as on the level of the whole organism. SAMHD1 plays a crucial role in shaping innate and adaptive immune responses. Since SAMHD1 has been demonstrated to efficiently inhibit a wide variety of viruses (52), many of these viruses, in turn, have developed various mechanisms to conquer SAMHD1 imposed block to viral replication (52, 63). Moreover, the NFκB signaling pathway has been shown to play a role in previous CoVs (72, 73). The fact that SAMDH1 has been shown to have proviral activities by suppressing the NFκB signaling pathway is significant. It is imperative to understand the full mechanisms in the pathogenesis of COVID 19, which may help develop new drugs and modulate SAMHD1 activity.

## ACE2 Localization In The Human Brain

### Evidence of Expression and Distribution of ACE2 in Brain

ACE2 that has been established as the functional receptor for SARS-CoV-2 is a transmembrane monocarboxypeptidase (8, 13, 74). It is significantly important to note that in addition to other human organs, kidneys, lungs, heart, and testes, it has been reported to be expressed in the brain in several databases as well as in the scientific literature (75). Initially, ACE2 was sequenced and cloned from the human failing heart and human lymphoma cDNA libraries (76, 77). The distribution of ACE2 in the brain was disputed in the beginning since original reports were unable to detect carboxypeptidase in this tissue (78). Later, studies of animal models exhibited the widespread presence of ACE2 mRNA and protein in the mouse brain (75). Animal model studies suggested that the ACE2 presence in nuclei has been identified in the central management of cardiovascular function including subfornical organ, paraventricular nucleus (PVN), the nucleus of the *tractus solitarius* (NTS), and rostral ventrolateral medulla (RVLM) (79, 80). The presence of ACE2 was also reported in non-cardiovascular areas, which included the motor cortex and raphe. Another study reported the occurrence of ACE2 mRNA and protein in the mouse brainstem (81). In addition, recent studies have also described the presence of ACE2 mRNA in the rat *medulla oblongata* and mouse brain (82, 83).

### ACE2, a Member of Brain RAS May Have Cardioprotective, and Cerebro-Protective Effects

It is well-established the fact that ACE2 is an elegant and integral member of the renin-angiotensin system (RAS) (84). In the body, RAS functions to regulate blood pressure, kidney function, and salt and water homeostasis (84). Importantly, there is a RAS in the brain, as well (85). Brain RAS is similar to the other tissue RAS (86). ACE2 has been reported to be expressed specifically in the areas controlling central blood arterial pressure (78). Several studies have revealed that brain ACE2 has been involved in the progression of neurogenic hypertension, the molecular mechanisms, by which this protective effect occurs, seem to include the regulation of angiotensin (AT) receptors expression. ACE2 is capable of adjusting the AT2/AT1 and Mas/AT1 ratios so that it opposing the promotion of hypertension. In addition, animal model demonstrated that nitric oxide (NO) signaling pathways may also be influenced by ACE2 over-expression in the CNS as demonstrated by an overall reduction of hypertension of neurogenic type in syn-hACE2 mice (78, 79, 84). Therefore, it has been suggested that since ACE2 possesses a monitoring role in the central regulation of cardiovascular function and blood pressure (86), ACE2 in the brain is a potential therapeutic target for hypertension and other cardiovascular diseases that result from an overactive RAS (80). This fact is significantly crucial in the context of COVID-19 because, apart from pneumonia, COVID-19 leads to acute myocardial injury and, progressively, chronic damage to the cardiovascular system (87). It is conceivable that cardiovascular protection is a vital part of treatment for COVID-19 (87, 88). Targeting ACE2 on the central regulation of cardiovascular function may at least partially be helpful in the treatment of COVID-19.

Also, ACE2 has a peripheral other than cardio-cerebral vascular protection effect (86, 89). The significance of peripheral ACE2 in regulating blood pressure is well-established, but fewer studies emphasize that ACE2 plays a key role in regulating blood pressure (15, 16, 90). Uncharacteristically elevated blood pressure increases the risk of cerebral hemorrhage (26). It is to note that death associated with cerebrovascular disease has been reported in patients suffering from COVID-19 (14). Activation of ACE2/Ang (1–7)/MASR axis has also been shown to improve neurological deficits as it has anti-inflammatory and antioxidative effects in the ischemic insult. Thus, ACE2/Ang (1–7)/MASR axis is considered to play a protective role in the treatment of ischemic stroke, as well as other cerebrovascular events (86). Currently, the neuroinvasive propensity of COVID19 is well established (8, 13, 28, 91, 92). Also, there is evidence from animal studies that ACE2 receptors are present in areas such as medulla and brainstem (82, 83). Therefore, as suggested previously, the pathogenesis SARS-CoV-2-induced respiratory failure may also partially stem from the brain.

### Long Term Complication of COVID 19 Invasion in the Brain

Although they are known as respiratory pathogens, the neuroinvasive and neurotropic properties of CoVs have been demonstrated in mice (93–95). Also, studies reveal that a human CoV (OC43) is capable of infecting and then persisting in human neuronal and glial cells, subsequently activating glial cells to deliver pro-inflammatory mediators and causing neurodegeneration (93–97). COVID-19 appears to be neuroinvasive using the ACE2 functional receptor (20–23). As mentioned above, ACE2 is an important component of RAS, and pathways associated with it have been correlated with Alzheimer disease as well as other neurodegenerative diseases (74, 81, 91). Dysregulated brain RAS has been associated with neurodegeneration due to the strong evidence for initiating a cascade of events leading to increase in oxidative stress, apoptosis, and neuroinflammation causing progressively neurodegeneration. Degenerative diseases of the brain that have been linked to altered RAS include Parkinson disease, Alzheimer disease, Huntington disease, multiple sclerosis, amyotrophic lateral sclerosis, traumatic brain injury, other than stroke as mentioned above (86, 98, 99). Moreover, the dysregulation of the antiviral response is another factor that has been associated with neurodegenerative disease (100, 101). It has been shown that products of certain genes may have potent antiviral activities, and may have deleterious effects when their expression is not appropriately regulated. There is evidence of mutations that have been linked to neurological conditions occurring in genes related to the antiviral response. Among such genes, SAMDH1 is probably the one. Notably, SAMDH1 has been related to Aicardi-Goutieres syndrome—a congenital disease that includes neurodegeneration as one of its main symptoms (62). As mentioned above, SAMDH1 appears to control the NF-κB pathway. Importantly, NF-κB has been engaged in the pathogenesis of a range of neurodegenerative disorders, including Alzheimer disease, Parkinson disease, Huntington disease, diabetic neuropathy, AIDS-dementia, and amyotrophic lateral sclerosis (102–104). Taken together, based on its similarity with other CoVs, and the fact that COVID 29 is neuroinvasive and its functional receptor is ACE2, and also because SAMDH1 is upregulated in the pathogenesis of COVID 19, it can be hypothesized that the SARS-CoV-2 entry into the brain will result in long-term neurological sequelae and may ultimately lead to neurodegenerative changes.

Although text mining and databases are useful to suggest mechanisms, there is one limitation of our study is that it relies on computational methods. Despite a sophisticated analysis was carried out, this study may not obviously predict the postulated interactions with 100% accuracy (42–45). Since the pandemic has been declared in March 2020, almost 19 million global cases and over 700,000 global deaths have been recorded on August 6, 2020. In these months several laboratories have also collected plasma as well as other body fluids that will be key in testing several hypotheses and confirm the presumed role of SAMDH1 in triggering the neurological symptoms of COVID 19 infection.

## Conclusions

There is accumulating evidence that COVID-19 involves the brain. Not only studies have reported mild neurological symptoms, but encephalitis and cerebrovascular disease have been reported. Most struggle in the human war against this new virus revolves around the fact that COVID 19 is a respiratory pathogen. Mortality associated with this virus is because of respiratory failure, cardiovascular damage, and cerebrovascular accidents. In this study, we have explored the role of SAMDH1 as the potential link between SARS-CoV-2 infection and neurological illness. We discussed the distribution of ACE2 in CNS and its vital role regulation of cardiovascular function and, specifically, blood pressure in the brain (centrally) and at the periphery. We assume that detailed neurological and neuropathological investigations are ongoing. Establishing a therapeutic strategy that targets the brain ACE2 may improve the outcome of COVID-19 patients. Interestingly, the same SAMDH1 has been associated with various neurological diseases, including Aicardi-Goutieres syndrome (AGS) as well as GBS, which has been associated with Zika Virus infection. We strongly suggest that SAMDH1-targeting COVID-19 may be at the basis of both early neurological symptoms and late neurodegeneration and our neuropathological team is open to any cooperation.

## Author Contributions

AK conceptualized the study, collected data, and drafted the initial manuscript. CS designed the study and revised the manuscript. All authors meet the ICMJE requirements for authorship, approved the final manuscript as submitted, and agree to be accountable for all aspects of the work.

## Conflict of Interest

The authors declare that the research was conducted in the absence of any commercial or financial relationships that could be construed as a potential conflict of interest.
